# Obstetric Fistula Is a “Neglected Tropical Disease”

**DOI:** 10.1371/journal.pntd.0001769

**Published:** 2012-08-28

**Authors:** L. Lewis Wall

**Affiliations:** 1 Department of Obstetrics & Gynecology, School of Medicine, Washington University in St. Louis, St. Louis, Missouri, United States of America; 2 Department of Anthropology, College of Arts and Sciences, Washington University in St. Louis, St. Louis, Missouri, United States of America

The term “neglected tropical diseases” (NTDs) is commonly used to refer to a “baker's dozen” of infectious conditions prevalent in resource-poor tropical countries. The 13 core diseases in the NTD group include ascariasis, dracunculiasis, hookworm, lymphatic filariasis, onchocerciasis, schistosomiasis, trichuriasis, Chagas disease, human African trypanosomiasis, Buruli ulcer, leprosy, and trachoma [1]. The World Health Organization has expanded the list to 17 conditions (http://www.who.int/neglected_diseases/en/), and some authors add another 20 or so fungal, viral, and ectoparasitic infections to the list [1]. Here I argue that obstetric fistula should be classified as an NTD because it, like the infectious conditions listed above, is also a preventable, treatable malady responsible for much suffering, stigmatization, and lost productivity among the impoverished “bottom billion” of the world's population.

The entities traditionally classified as NTDs all have an infectious etiology. They are caused by exotic pathogens that flourish largely (but not exclusively) in tropical climates. Although this common biological underpinning is important, it should not cause us to overlook other, non-infectious pathologies that are prevalent in poor countries and which also produce enormous human suffering, because not all human pathology is infectious in origin and not all human suffering can be treated with antimicrobial agents.

In clinical practice, diseases are manifested as specific *illnesses*, uniquely experienced by individual patients. Because much human illness is actually due to derangements in physiological functioning rather than the tissue changes traditionally studied by pathologists under the microscope or what a culture plate grows in a microbiology lab, medical textbooks are shifting their perspective towards discussion of “disorders” of various organ systems rather than limiting themselves to the more traditional discussion of “diseases.” We need to think of NTDs not as neglected tropical *diseases* but rather as neglected tropical *disorders*, recognizing that although they occur primarily in the tropics they are often linked more directly to poverty, economic insecurity, social instability, and institutional mismanagement than they are to climatic conditions. The classification of podoconiosis—a debilitating form of elephantiasis that mimics the classical infection produced by *Wuchereria bancrofti* (but which actually is an inflammatory geochemical disorder caused by silica particles absorbed through the feet of susceptible, barefooted farmers who are chronically exposed to volcanic red-clay soils)—as an NTD is evidence of this type of paradigm shift [2], [3].

Obstetric fistula is a striking example of a neglected tropical disorder of this kind. Once prevalent in Western countries, obstetric fistula is today largely confined to the cultures of tropical poverty. Obstetric fistulas are caused by obstructed labor. Labor is obstructed when the fetus will not fit through the birth canal during the second stage of labor. This occurs either because the pelvis is too small, the baby is too large, or a malpresentation renders normal obstetrical mechanics impossible [4]. Because it is an involuntary process, once labor starts, the uterus contracts until its contents have been expelled, the uterus ruptures, or the laboring woman dies. When labor is obstructed, the presenting part of the fetus (usually the fetal head) is wedged progressively deeper into the pelvis until its further advance is prevented by the presence of the unyielding bones of the pelvic girdle. The uterine contractions relentlessly compress the entrapped soft tissues of the bladder, vagina, and pelvis between the two opposed boney surfaces, eventually shutting off their blood supply. The entrapped tissues die and slough away, creating a fistula between the urinary tract and the vagina (and sometimes between the rectum and the vagina as well) through which urine (or stool) leaks in a continuous and unremitting stream (Figure 1).

**Figure 1:**
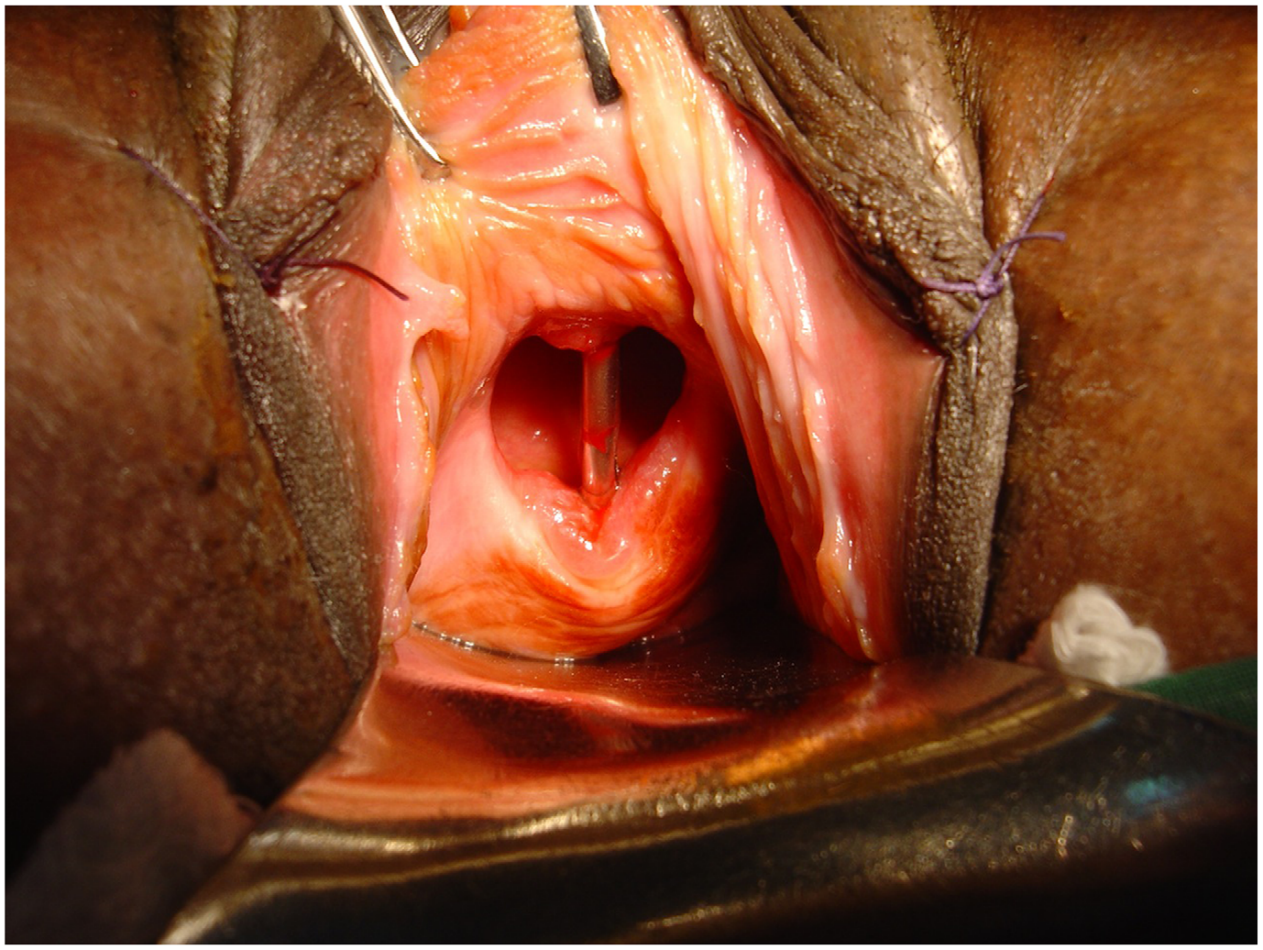
Obstetric vesico-vaginal fistula from prolonged obstructed labor. Transvaginal view of a fistula produced by obstructed labor. Prolonged pressure from the entrapped fetal head has interrupted the blood supply to the vesico-vaginal septum, resulting in tissue necrosis and formation of a fistula. A metal catheter placed into the bladder through the urethra is clearly visible through the defect in the bladder base. The fistula causes continuous and uncontrollable loss of urine, which can only be corrected by surgery. (Photograph courtesy Dr. Andrew Browning.)

Obstetric fistulas are completely preventable—if obstructed labor is diagnosed early and if appropriate intervention occurs in a timely fashion. This requires the presence of a skilled attendant at birth who can make the correct diagnosis and a system of management that functions efficiently to make sure needed interventions take place before the awful consequences of prolonged obstructed labor develop. Treating obstructed labor correctly often requires surgery: performance of a cesarean section that bypasses the obstruction in the pelvis and creates a new trans-abdominal passageway through which the fetus is extracted. Where skilled attendance at birth is lacking, however, a woman may linger in labor for up to a week. The suffering this entails is almost unimaginable to those living in resource-rich countries. When obstructed labor is protracted, fetal mortality almost always exceeds 90% [4]. After a day or two of unendurable stress, the entrapped fetus dies from asphyxiation. As the tissues decay, the fetus gradually macerates within the birth canal, eventually softening enough to allow delivery—unless the uterus ruptures from the unrelenting contractile activity or the woman dies from hemorrhage, infection, exhaustion, dehydration, or the combination thereof. To the agony of prolonged labor is added the grief of a stillborn child, yet the worst is often yet to come.

Women who survive the ordeal of obstructed labor only to develop an obstetric fistula are often doomed to a life of utter misery. The woman with an obstetric vesicovaginal or rectovaginal fistula becomes an outcast. Unclean, soiled, stinking, continually wet, and stigmatized by communities that do not understand that fistulas are the result of faulty obstetrical mechanics and not immoral personal behavior, afflicted women are usually divorced by their husbands and often are cast out by their families, relegated to precarious lives on the margins of society [5]. If a woman survives obstructed labor and develops a fistula, she will likely live for many years, since the condition by itself is not fatal. Still worse, because obstructed labor produces a field injury that may involve many different pelvic organ systems, she may also have other debilitating comorbidities along with her urinary or fecal incontinence: foot-drop from lumbo-sacral nerve damage, vaginal stenosis and infertility, pelvic pain, chronic vulvar dermatitis from constant maceration of the skin in urine and feces, depression, social isolation, and post-traumatic stress disorder [4], [6]. Sometimes the end result is suicide.

Obstetric fistulas from prolonged obstructed labor were common in Western countries 150 years ago, but now they are long gone. The eradication of obstetric fistula from the affluent world is a triumph of modern obstetrics, yet some 3.5 million women in resource-poor nations remain afflicted with this terrible condition [4]. The occurrence of an obstetric fistula in Europe or North America is unusual enough to merit publication as a case report in a medical journal [7], but as many as 130,000 new cases occur each year in Ethiopia, Uganda, Niger, Nigeria, Afghanistan, Sierra Leone, and other parts of sub-Saharan Africa and south Asia [4]. Because the condition is not fatal and because the institutional capacity to provide reconstructive services is so poor, the worldwide burden of women with obstetric fistula increases every year.

This is a classic example of a neglected tropical disorder that shares all of the characteristics common to neglected tropical infectious diseases, except an infectious etiology [1]. It is rarely seen in developed nations. It primarily affects the poor and so perpetuates (if not increases) poverty among those afflicted. It is under-appreciated as a cause of morbidity and mortality. It is highly stigmatizing. It can be treated or controlled using proven, cost-effective means, but the necessary resources have not been mobilized for the benefit of the affected populations. Most importantly, however, unlike the other NTDs, obstetric fistula affects women exclusively. Here the burden produced by pathophysiology is increased dramatically by gender inequity, asymmetries of social, political, and economic power, and by medical institutions that fail to deliver high quality reproductive health care to the populations that need it most.

Obstetric fistula vanished from industrialized nations because Western women get prompt emergency obstetric care when they need it. Because obstructed labor is one of the five main causes of maternal death worldwide, the incidence and prevalence of obstetric fistula closely tracks maternal mortality ratios [6]. When maternal mortality fell precipitously throughout the Western world during the 20th century, the obstetric fistula also vanished [8]. In the cultures of tropical poverty, however, the infrastructure needed to provide high quality maternal health care is poorly developed. This neglect is a reflection of the inferior status held by women and the resulting lack of political influence that they hold in these countries [9], [10]. Where fistulas are prevalent and maternal death is common, rates of skilled attendance at birth and rates of cesarean delivery are usually far below the most minimal standards required to preserve maternal health [11]. Furthermore, access to skilled obstetric services such as cesarean section is usually available only to the affluent classes of urban society, whereas the need for such care is greatest among the poor, especially in rural areas [12].

Obstetric fistula is unique among the neglected tropical disorders in that both its prevention and its treatment are surgical. Obstetrics is a surgical specialty, whether it involves instrumental vaginal delivery, cesarean section, or repair of a traumatic birth injury. This is another reason why fistula is neglected: it falls outside the familiar paradigm common to public health and infectious disease control. The provision of surgical services has not generally been a priority in tropical medicine, yet in many cases surgery plays a vital role in caring for those afflicted with the traditional NTDs [13]. Wound care is important in Buruli ulcer. Wound care and tendon transfers can restore lost function in patients with leprosy. Eyelid surgery in trachoma patients can prevent blindness. Patients with scrotal lymphedema from lymphatic filariasis can be restored by hydrocele surgery. And successful obstetric fistula repair is transformative: it can pull a teenaged girl back from the abyss of despair and give her a new life.

Increasing the emphasis on neglected tropical *disorders*—including obstetric fistula— should include the creation of centers that provide basic surgical services to those most afflicted by these conditions. It is no surprise, for example, that The Worldwide Fistula Fund developed its new obstetric fistula surgery center in Danja, Niger, on the grounds of a leprosy hospital that also provides wound care and ophthalmologic surgery (http://www.worldwidefistulafund.org). Thought should be given to dismantling barriers between disease categories, creating new therapeutic synergies, and developing more effective institutions for health care delivery. If fistula centers throughout Africa began to offer hydrocele surgery to men with lymphatic filariasis and worked to establish reciprocal programs for women in places where the emphasis has been largely on problems of male urology, further barriers could be eliminated.

We should move beyond the limitations imposed by considering only infectious agents as the cause of neglected tropical disease and pay more attention to the structural forces that produce human suffering whatever the geographical location of the patients [14]. The burden of neglected tropical diseases is not simply a reflection of exotic biology. It is also a reflection of failed political institutions, misplaced priorities, and socioeconomic structures that favor entrenched elites at the expense of the poor and marginalized [15]. Women are among the most prominent victims of these conditions, and the persistence of obstetric fistula into the 21st century is a stunning indictment of the world's failure to provide essential reproductive health care to those most in need of it. Safe childbirth is a basic human right; the challenge is to make it a practical reality for everyone.

## References

[1] Hotez PJ, Fenwick A, Savioli L, Molyneux DH (2009) Rescuing the bottom billion through control of neglected tropical diseases. Lancet 373: 1570–1575. 19410718 10.1016/S0140-6736(09)60233-6

[2] Davey G, Newport M (2007) Podoconiosis: the most neglected tropical disease? Lancet 369: 888–889. 17368134 10.1016/S0140-6736(07)60425-5

[3] Ayele FT, Adeyemo A, Finan C, Hailu E, Sinnott P, et al (2012) HLA class II locus and susceptibility to podoconiosis. N Engl J Med 366: 1200–1208. 22455414 10.1056/NEJMoa1108448 PMC3350841

[4] Wall LL (2006) Obstetric vesicovaginal fistula as an international public health problem. Lancet 368: 1201–1209. 17011947 10.1016/S0140-6736(06)69476-2

[5] Wall LL (2002) *Fitsari 'dan Duniya*: an African (Hausa) praise-song about vesico-vaginal fistulas. Obstet Gynecol 100: 1328–1332. 12468180 10.1016/s0029-7844(02)02498-5

[6] Arrowsmith S, Hamlin EC, Wall LL (1996) Obstructed labor injury complex: obstetric fistula formation and the multifaceted morbidity of maternal birth trauma in the developing world. Obstet Gynecol Surv 51: 568–574. 8873157 10.1097/00006254-199609000-00024

[7] Korell AN, Argenta PA, Strathy JH (2007) Prolonged obstructed labor causing a severe obstetric fistula: a case report. J Reprod Med 52: 555–556. 17694983

[8] Loudon I (1992) The transformation of maternal mortality. Lancet 305: 1557–1560. 10.1136/bmj.305.6868.1557 PMC1884697 1286386

[9] Weil O, Fernandez H (1999) Is safe motherhood an orphan initiative? Lancet 354: 940–943. 10489970 10.1016/S0140-6736(99)02369-7

[10] Kyomuhendo GB (2003) Low use of rural maternity services in Uganda: impact of women's status, traditional beliefs, and limited resources. Reprod Health Matters 11 (21) 16–26. 12800700 10.1016/s0968-8080(03)02176-1

[11] Dumont A, de Bernis L, Bouvier-Colle MH (2001) Bréart G; MOMA Study Group (2001) Caeserean section rate for maternal indication in sub-Saharan Africa: a systematic review. Lancet 358: 1328–1334. 11684214 10.1016/s0140-6736(01)06414-5

[12] Ronsmans C, Holtz S, Stanton C (2006) Socioeconomic differentials in caesarean rates in developing countries: a retrospective analysis. Lancet 368: 1516–1523. 17071285 10.1016/S0140-6736(06)69639-6

[13] Ozgediz D, Riviello R (2008) The ‘other’ neglected diseases in global public health: surgical conditions in sub-Saharan Africa. PLoS Med 5: e121 doi:10.1371/journal.pmed.0050121. 18532875 10.1371/journal.pmed.0050121 PMC2408612

[14] Farmer P (2003) Pathologies of power: health, human rights, and the new war on the poor. Berkeley: University of California Press.

[15] Acemoglu D, Robinson J (2012) Why nations fail: the origins of power, prosperity, and poverty. New York: Crown Business-Random House.

